# Primary Carcinosarcoma of the Pancreas With CD10-Positive Sarcoma Component

**DOI:** 10.1177/2324709617740906

**Published:** 2017-11-06

**Authors:** Christine J. Salibay, Julia Rewerska, Shweta Gupta, Nicholas Ree

**Affiliations:** 1 University of Illinois at Chicago Hospital and Health Sciences System, Chicago, IL, USA; 2 John H Stroger Jr. Hospital of Cook County, Chicago, IL, USA

**Keywords:** carcinosarcoma, pancreatic, adenocarcinoma, primary pancreatic

## Abstract

Carcinosarcomas of the pancreas are rare entities with a dismal prognosis. We report a rare case of pancreatic carcinosarcoma in a 49-year-old African American female who underwent a total abdominal hysterectomy with right salpingo-oophorectomy and exploration of the pancreatic mass. The surgery revealed a sclerotic mass in the body and tail of the pancreas that was surgically unresectable, and a pancreatic biopsy confirmed the pathology of pancreatic carcinosarcoma. Histologically, the lesion showed a high-grade spindle cell sarcoma and adjacent moderately differentiated adenocarcinoma. On immunohistochemical examination, the carcinomatous component was positive for epithelial markers, and the sarcomatous component was focally positive for SMA and desmin. In addition, the sarcomatous component showed diffuse immunoreactivity for CD10 with a surrounding myofibroblastic proliferation. Reports have associated expression of CD10 in pancreatic stellate cells with increased tumor aggressiveness. In this article, we report a case of pancreatic carcinosarcoma that shows sarcomatous CD10 immunoexpression with higher Ki67 labeling in the sarcoma than the carcinoma raising the question if the sarcomatous component could be potentiating the aggressiveness of the carcinomatous component.

## Introduction

Carcinosarcomas arising in the pancreas are exceedingly rare with few cases reported in the literature and with the existence of primary pancreatic carcinosarcoma having been only recently acknowledged.^1,2^ Differential diagnosis for these cases include sarcomatoid carcinoma of the pancreas, an entity most consistent with poorly differentiated carcinoma showing immunoreactivity to cytokeratins in sarcomatous components.^1-3^ We discuss a case of a patient with pancreatic carcinosarcoma composed of a moderately differentiated adenocarcinoma and a high-grade sarcoma, bilateral mature ovarian teratomas, and a fibroid uterus.

## Case Report

The patient is a 49-year old African American female G2P2002. The patient’s medical history is significant for hypertension and notably on an angiotensin receptor blocker, long-term cigarette smoker, pancreatitis, and mature cystic teratoma of the left ovary status post left oophorectomy a month prior to referral. The patient presented with persistent abdominal and back pain and with an 85-pound weight loss within 1 year. Imaging showed masses in the body/tail of the pancreas and uterus/right adnexa. Serum markers were within normal limits, except a minimally elevated human chorionic gonadotropin of 20 pg/mL. Given the possibility of a uterine or right adnexal primary, a total abdominal hysterectomy with right salpingo-oophorectomy and exploration of the pancreatic mass were performed. The surgery revealed a sclerotic mass in the body and tail of the pancreas that grossly involved the superior mesenteric artery (unresectable); therefore, the tumor was left in situ and pancreatic biopsy was taken for histopathology.

**Figure 1.**
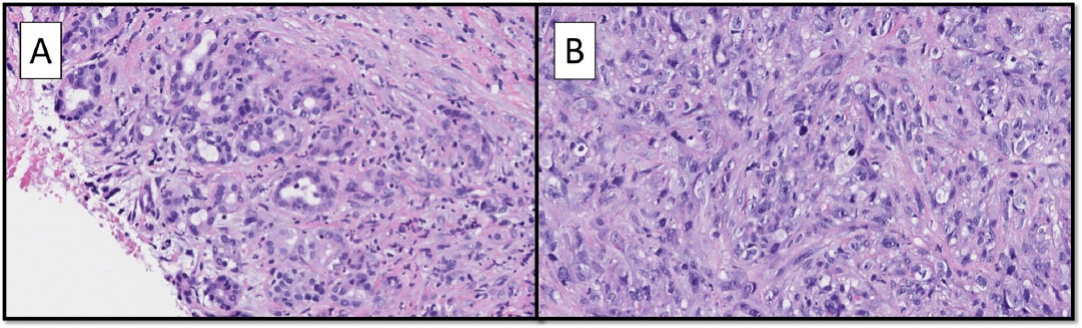
(A) The tumor was composed of an adenocarcinoma component (hematoxylin-eosin; magnification 200×). (B) An atypical sarcomatous component (hematoxylin-eosin; magnification 200×).

**Figure 2.**
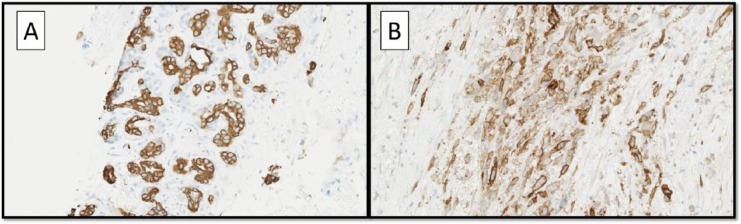
(A) Adenocarcinoma stained strongly for AE1/AE3 with negative stroma around the tumor (AE1/AE3 immunostain; magnification 200×). The stromal component was positive for (B) CD10 (CD10 immunostain; magnification 200×).

The hysterectomy specimen revealed a fibroid uterus. The mass adjacent to the right ovary was a benign subserosal uterine polypoid leiomyoma. The right cystic ovary was entirely submitted for histologic evaluation and was a mature teratoma. Histology of the pancreatic biopsy showed a high-grade spindle cell sarcoma and adjacent moderately differentiated adenocarcinoma (Figure 1A and B). Around the sarcoma were a concentration of myofibroblasts marking with desmin and smooth muscle actin (SMA), and overall the background tissues were prominently fibrotic. Additionally, the hepatocholedochal lymph node submitted showed a focus of metastatic adenocarcinoma of 0.85 mm. Immunohistochemical stains were performed on the current and prior biopsy specimens (Table 1). The adenocarcinoma component was, on immunohistochemistry, compatible with a pancreatic primary (Figure 2A). The sarcoma stained focally for SMA, focally for desmin, and showed strong diffuse staining with CD10 (Figure 2B). The diagnosis of pancreatic carcinosarcoma was made.

**Table 1.** Immunohistochemical Results of Adenocarcinoma and Sarcoma Components.

	Adenocarcinoma	Sarcoma
CD10	−	+
CDX2	+ (f)	n/a
Cytokeratin AE1+3	+	−
Cytokeratin 7	+	n/a
Cytokeratin 20	−	n/a
Desmin	−	+ (f)
Estrogen receptor	−	−
Myogenin	−	−
P53	Within normal limits	Within normal limits
SMA		+ (f)
TTF1	−	n/a
Villin	+	−
Ki67	50%	90%
DOG1	n/a	−
CD117	n/a	−

Abbreviations: (f), focal; +/−, weakly positive; SMA, smooth muscle actin; TTF-1, thyroid transcription factor-1; n/a, not applicable.

After discussion in the multidisciplinary tumor board, the patient received chemotherapy with gemcitabine and docetaxel with no response, followed by ifosfamide and Adriamycin with progression. After failing these 2 lines of chemotherapy, palliative radiation therapy was planned; however, the disease rapidly progressed and the patient died approximately 10 months postdiagnosis.

## Discussion

Several theories hypothesize the histogenesis of carcinosarcomas, although it remains unclear.^4,5^ The “collision” theory indicates 2 distinct neoplastic entities arising from 2 independent neoplasms.^2,4^ “Combination” describes a tumor from one stem cell line differentiating into epithelial and mesenchymal components.^2,4^ “Transformation” describes a metaplastic change in a neoplasm resulting in both components.^2,4^ As an example, for so called carcinosarcomas (malignant mixed mullerian tumors) of the uterus, an epithelial to mesenchymal transition in which the carcinoma transitioned to a sarcomatous component is hypothesized for most cases because cytokeratin is positive in both the carcinomatous and sarcomatous components, albeit often focal in the later. Also, Ki67 labeling studies on malignant mixed mullerian tumors show the carcinomatous component to have a higher Ki67 labeling than the sarcomatous component, suggesting that the sarcomatous component is derived from the carcinoma.^6,7^ In contrast to malignant mixed mullerian tumor, the carcinomatous and sarcomatous components of the pancreatic carcinosarcoma in this case showed very different immunohistochemical staining profiles with the sarcomatous component having a higher Ki67 rate of approximately 90% versus the carcinomatous component with a rate of approximately 50%.

In the literature, immunohistochemical and molecular analyses favor a monoclonal origin for pancreatic carcinosarcoma. Kim et al discovered identical mutations of *Kras* gene accompanied with a strong nuclear p53 immunoreactivity in both sarcomatous and carcinomatous components indicating a monoclonal origin supporting metaplastic transformation of a carcinoma.^8^ More recently, Bai et al investigated the origin of pancreatic carcinosarcomas and their findings were similar to earlier studies showing strong nuclear p53 immunoreactivity and a similar *Kras* gene mutation in both components, favoring the hypothesis of metaplastic transformation of a carcinoma.^4^ In contrast, the pancreatic carcinosarcoma of this case displayed wild type p53 in both the carcinomatous and sarcomatous components, and the immunohistochemical profiles of the 2 components were very different. This would argue against a monoclonal origin. Also, the monoclonal origin hypothesis would be more in line with a sarcomatoid carcinoma rather than a true carcinosarcoma.

CD10 is a zinc-dependent metalloproteinase. Recent research has been focusing on the significance of stromal cells in the background of carcinomas. Within the pancreas, it is the pancreatic stellate cell that has been of interest. Pancreatic stellate cells that express CD10 are associated with more aggressive pancreatic carcinomas and increased metalloproteinease 3 enzyme production, which is involved in basement membrane degradation and, therefore, tumor invasiveness.^9^ Interestingly, the sarcomatous component of the pancreatic carcinosarcoma from this case was strongly and diffusely positive for CD10. It has been proposed that CD10 could be a target for treatment of pancreatic carcinomas using metalloproteinase inhibitors. As an example, fish oil, which has among its effects metalloproteinase inhibiting properties, has shown benefit as reported in the literature.^10-17^ However, the stromal biology of cancers is complex and care in manipulating the tumor stroma is needed as cancer stage, patient selection, and possibly proper timing with chemotherapy may make a polar difference in prognosis.^18^ As pancreatic carcinosarcomas are rare, no known reports have evaluated for CD10 positivity or mentioned use of any metalloproteinase inhibiting substances, which will need further study.

In conclusion, reported is a case of pancreatic carcinosarcoma composed of a moderately differentiated adenocarcinoma with metastasis, and a high-grade sarcoma component that showed strong diffuse CD10 positivity. We demonstrate the sarcomatous component of this case of pancreatic carcinosarcoma to have a higher Ki67 proliferation rate than the carcinomatous component and show immunophenotypic similarities between the sarcomatous component and pancreatic stellate cells. This raises the hypothesis if the sarcomatous component could potentiate the aggressiveness of the carcinomatous component in a similar mechanism as the pancreatic stellate cells do.
